# Dermatoglyphic Patterns in Cystic Fibrosis Children

**Published:** 2014-09-12

**Authors:** Atefeh Ezzati, Fereshteh Batoei, Seyed-Ali Jafari, Mohammad-Ali Kiyani, Naser Mahdavi-Shahri, Hamid Ahanchian, Shahrzad Tehranian, Hamid-Reza Kianifar

**Affiliations:** 1 Clinical Research Development Center, Ghaem Hospital; 2 Mashhad University of Medical Sciences; 3 Department of Pediatric Gastroenterology; 4 Department of Biology, Faculty of Sciences, Ferdowsi Institute of Biotechnology; 5 Nuclear Medicine Research Center, Mashhad University of Medical Sciences, Mashhad, Iran

**Keywords:** Dermatglyphics, Cystic Fibrosis, Palm Patterns, Fingerprints, Children

## Abstract

***Objective:*** It is believed that fingerprints and palm patterns may represent genetically determined congenital abnormalities in Cystic Fibrosis (CF). The main idea of this paper was to determine differences of fingerprints and palm patterns in CF and normal children.

***Methods:*** Forty-six CF children (27 males, 19 females) and 341 (113 males, 228 females) healthy individuals were recruited for this study. Fingerprint patterns, Total ridge count (TRC) of each finger, a-b ridge count, and atd angles of all participants were recorded. Asymmetry of the right and left hand for each value was determined and dissimilarity in fingerprint patterns between homologous fingers was compared using Chi-square analysis, Mann-Whitney U test and Fisher's exact test.

***Findings:*** There were significant differences in the mean TRC of the right digit IV (*P*=0.009), left digit III (*P*=0.02), left digit IV (*P*=0.03), and left digit V (*P*=0.03). Furthermore, we found significant differences in right atd angel (*P*=0.001), left atd angel (*P*=0.002), right a-b ridge (*P*=0.007) and left a-b ridge (*P*=0.001). In contrast, we found no significant differences in atd angle asymmetry, a-b ridge count asymmetry and pattern dissimilarity score between both groups (*P*>0.05).

***Conclusion:*** Dermatoglyphic characteristics could be used as a supplementary diagnostic method in CF children.

## Introduction

Dermatoglyphics is the science of fingerprints and palm prints analysis which are constant throughout life^[^^1^^-^^3^^]^. It is believed that they may represent different genetically determined congenital abnormalities. Several genes are involved in the inheritance of the dermal traits^[^^1^^]^. Dermal ridges form in 6^th^ week of gestation and reach their maximum size between 12^th^ and 13^th^ week. Historically, dermatoglyphics was a classical model to consider the polygenic inheritance. Therefore, some specific dermatoglyphic patterns may be accompanied with different genetic abnormalities^[^^1^^,^^3^^]^.

 Recent studies on dermatoglyphics have shown especial patterns in various congenital abnormalities such as skeletal maturation, diabetes type 1, schizophrenia, albinism, cleft lip and palate, rheumatoid arthritis, congenital spinal cord anomalies and Klinefelter syndrome^[^^4^^-^^10^^]^.

 Cystic Fibrosis (CF) is a common fatal, autosomal recessive disease in Caucasians^[^^11^^,^^12^^]^. CF is a progressive disease that involves exocrine glands, lungs, gastrointestinal system, pancreas, liver, kidneys and reproductive system^[^^3^^,^^12^^,^^13^^]^. CF prevalence varies from 1 to 2500 among Caucasians^[^^13^^]^; 10 million carriers of the recessive gene exist in the world^[^^14^^]^. CF can be diagnosed antenatally using genetic tests and in early childhood with sweat test^[^^15^^]^.

 The main idea of this paper was to determine the differences in dermatoglyphic patterns in CF and normal children.

## Subjects and Methods

This is a case-control study performed between October 2012 and March 2013. The study group included 46 children with CF, confirmed by two positive sweat tests, referred to CF clinic in Dr. Sheikh Pediatric Hospital, Mashhad University of Medical Sciences, Iran. The control group consisted of 341 healthy participants. Exclusion criteria included children less than one year of age, individuals with current atopic dermatitis and any finger or palm abnormalities which interfered with the procedure. All subjects were informed about the process and consent forms were obtained.

 We used red powder blush to roll finger and palm separately. The powder was rubbed on palm and fingers of both hands by means of a brush. A piece of adhesive tape was placed on a rolling plate and the participant pressed his/her palm on the tape in distal to proximal direction. It was then transformed onto a labeled paper indicating right and left side. The same process was carried out for fingerprints as each finger was pressed separately to the adhesive tape. Fingers were named with roman numbers from I (thumb) to V (little finger). 

**Fig. 1:**
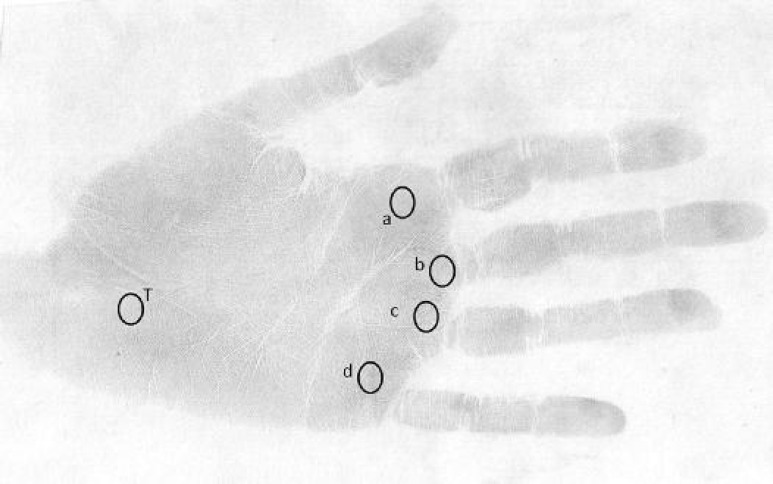
Palm patterns are labeled a, *b*, c, d and t

 Fingerprint pattern types were categorized into three groups, arch (A), loop (L) and whorl (W). Whorl was divided into two subgroups named simple whorl (W) and double whorl (2W) (Fig. 1). Patterns of homolog fingers in right and left hand were given 0 or 1 score due to their similarity or dissimilarity. Finally, these scores were added and final scores ranged 0 to 5^[^^2^^]^.

 After the center point of each fingerprint was joined to triradius (i.e. the conjunction point of three opposing ridge systems), TRC was calculated by summing the total numbers of ridges between the two centers^[^^16^^]^.

 Total ridge count of arch pattern is always zero because it has no triradius. Loop pattern has one triradius, but whorl pattern has two triradii. The higher calculated number of lines was reported for whorl pattern TRC (Fig. 1).

**Fig. 2:**
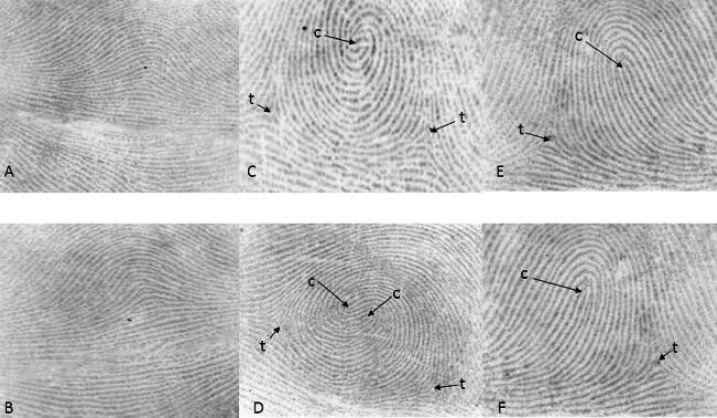
Fingerprint pattern types are categorized into three groups: arch (A,B), loop (F,G) and whorl. Whorls are divided into two subgroups named simple whorl (C) and double whorl (D). c: center point of fingerprint, t: triradius

 On the following step, we found the triradii (a, b, c, d) at the base of each finger (Fig. 2). a-b line was formed by joining a to b triradius.

a: triradius "a" at the base of the 2^nd^ finger

b: triradius "b" at the base of the 3^rd^ finger

c: triradius "c" at the base of the 4^th^ finger

d: triradius "d" at the base of the 5^th^ finger

t: triradius "t" at the proximal part of the palm

The ridges on the a-b line in both hands were calculated with magnifying glass.

 After detection of triradius a (at the base of 2^nd^ finger on palm print), t (on the proximal of palm) and d (at the base of 5^th^ finger on the palm), atd angle was drawn and measured by protractor in both hands.

 All the data was collected twice by one person and the mean of these two numbers was finally reported.

 We used chi-square test for comparing frequency of pattern types, Fisher's exact test for comparing the fingerprint pattern types and the disease phenotype and Mann-Whitney test to compare a-b ridge count, TRC, atd angle and asymmetry of right and left hand between control and study groups. *P*-values less than 0.05 were considered statistically significant.

## Findings

A total of 387 participants, 46 CF and 341 healthy children were enrolled in this study. Table 1 shows the baseline characteristics of studied group.

 Number and percentage of disease phenotype(failure to thrive, steatorrhea, clubbing, liver disease, other gastrointestinal symptoms and respiratory symptoms) and dermatoglyphic pattern types of the studied group are shown in Table 2. There was no significant difference in dermatoglyphic pattern types and phenotype.

**Table 1:** Baseline characteristics of the studied groups

**Variable**		**Mean (SD)**	**Variable**	**n (%)**
**Age (month)**		105.9 (43.0)	**Relative parents**	30 (85.7)
**Weight**		22.49 (8.8)	**FTT**	16 (57.1)
**Height (cm)**		116.4 (20.0)	**Steatorrhea**	13 (46.4)
**HC** a ** (cm) **		50.98 (1.9)	**Clubbing**	1 (3.6)
**MAC** b **(cm)**		16.4 (2.3)	**Respiratory symptoms** c	15 (53.6)
**Sweat test**	**Cl **	101.7 (38.8)	**Other gastrointestinal symptoms** d	17 (60.7)
	**Na**	104.7 (47.6)		
**Age of onset (month)**		3.42 (6.29)	**Liver disease**	2 (7.1)
**Age at diagnosis (month)**		11.8 (17.2)		

a :Head Circumference

b : Mid arm circumference

c : Other gastrointestinal symptoms include vomiting, GER, rectal prolapse, chronic diarrhea

d : Respiratory symptoms include pneumonia, cough, wheezing, dyspnea

**Table 2:** Comparing the number and percentage of disease phenotype and dermatoglyphic pattern types in CF patients

**Digit**	**Pattern type**	**FTT **		**Steatorrhea**		**Clubbing**		**Liver disease**		**Other gastrointestinal symptoms ** a		**Respiratory symptoms ** b	
		**Right hand** **n (%)**	**Left hand** **n (%)**	**Right hand** **n (%)**	**Left hand** **n (%)**	**Right hand** **n (%)**	**Left hand** **n (%)**	**Right hand** **n (%)**	**Left hand** **n (%)**	**Right hand** **n (%)**	**Left hand** **n( %)**	**Right hand** **n (%)**	**Left hand** ** n (%)**
**I**	**Loop**	9 (60)	9 (75)	8(53.3)	6 (50)	0(0)	1(100)	1(6.7)	0(0)	10(66.7)	5(41.7)	5(33.3)	5 (41.7)
	**Arch**	___	0 (0)	___	0 (0)	__	0(0)	__	0(0)	__	0(0)	__	1 (100)
	**Whorl**	7 (53.8)	7 (46.7)	5 (38.5)	7 (46.7)	1(100)	0(0)	1(7.7)	2(13.3)	7(53.8)	12(80)	10(76.9)	9 (60)
	***P. *** **value**	0.7	0.17	0.43	1	0.46	0.46	1	0.52	0.7	0.05	0.05	0.4
**II**	**Loop**	5 (50)	8 (57.1)	4 (40)	7 (50)	0(0)	1(7.1)	1(10)	1(7.1)	6(60)	9(64.3)	6(60)	6 (42.9)
	**Arch**	2 (100)	2 (66.7)	1 (50)	1(33.3)	0(0)	0(0)	0(0)	0(0)	0(0)	0(0)	1(50)	2 (66.7)
	**Whorl**	9 (56.3)	6 (54.5)	8 (50)	5(45.5)	1(6.3)	0(0)	1(6.3)	1(9.1)	11(68.8)	8(72.7)	8(50)	7 (63.6)
	***P. *** **value**	0.5	1	0.84	1	1	1	1	1		0.05	0.8	0.5
**III**	**Loop**	12 (63.2)	9 (52.9)	8 (42.1)	6(35.3)	1(100)	1(100)	2(10.5)	2(11.8)	12(63.2)	10(58.8)	9(47.4)	7 (41.2)
	**Arch**	2 (100)	3 (75.0)	1 (50)	2(50)	0(0)	0(0)	0(0)	0(0)	0(0)	1(25)	1(50)	3 (75)
	**Whorl**	2 (28.6)	4 (57.1)	4 (57.1)	5(71.4)	0(0)	0(0)	0(0)	0(0)	5(71.4)	6(85.7)	5(71.4)	5 (71.4)
	***P. *** **value**	0.13	0.8	0.82	0.31	1	1	0.64	0.68	0.2	0.16	0.6	0.2
**IV**	**Loop**	8 (61.5)	7 (58.3)	6 (46.2)	4(33.3)	1(7.7)	1(8.3)	1(7.7)	1(8.3)	7(53.8)	7(58.3)	8(61.5)	7 (58.3)
	**Arch**	1 (100)	1 (100)	0 (0)	0 (0)	0(0)	0(0)	0(0)	0(0)	0(0)	0(0)	0(0)	0 (0)
	**Whorl**	7 (50)	8 (53.3)	7 (50)	9 (60)	0(0)	0(0)	1(7.1)	1(6.7)	10(71.4)	10(66.7)	7(50)	8 (53.3)
	***P. *** **value**	0.8	1	1	0.25	0.5	0.4	1	1	0.2	0.42	0.5	0.8
**V**	**Loop**	9 (50)	8 (50	6 (33.3)	6(37.5)	1(100)	1(6.3)	2(11.1)	1(6.3)	11(61.1)	9(56.3)	10(55.6)	11 (68.8)
	**Arch**	___	1 (100)	___	0(0)	__	0(0)	__	0(0)	__	0(0)	__	0 (0)
	**Whorl**	7 (70)	7 (70)	7 (70)	7(70)	0(0)	0(0)	0(0)	1(10)	6(60)	7(70)	5(10)	4 (40)
	***P. *** **value**	0.4	0.53	0.11	0.16	1	1	0.52	1	1	0.4	1	0.1

a : other gastrointestinal symptoms include vomiting, GER, rectal, Chronic diarrhea

b : respiratory symptoms include Pneumonia, Cough, wheezing, dyspnea

**Table 3:** Number and percentage of dermatoglyphic patterns in CF patients and control group

**Digit**	**pattern type**	**Right hand**			**Left hand**		
		**CF group** **n (%)**	**Control ** **n (%)**	**Total (%)**	**CF group** **n (%)**	**Control ** **n (%)**	**Total (%)**
**I**	**Loop**	25 (54.3)	162 (48.1)	187 (48.8)	24 (52.2)	173 (52.0)	197 (52.0)
	**Arch**	2 (4.3)	8 (2.4)	10 (2.6)	2 (4.3)	17 (5.1)	19 (5.0)
	**Whorl**	19 (41.3)	167 (49.6)	186 (48.6)	20 (43.5)	143 (42.9)	163 (43.0)
	***P*** **. value**	0.5			1		
**II**	**Loop**	21 (45.7)	162 (48.1)	183 (47.8)	24 (52.2)	159 (47.7)	183 (48.3)
	**Arch**	5 (10.9)	29 (8.6)	34 (8.9)	6 (13.0)	28 (8.4)	34 (9.0)
	**Whorl**	20 (43.5)	146 (43.3)	166 (43.3)	16 (34.8)	146 (43.8)	162 (42.7)
	***P.*** ** value**	0.9			0.4		
**III**	**Loop**	32 (69.6)	222 (65.9)	254 (66.3)	29 (63.0)	212 (63.5)	241 (63.4)
	**Arch**	4 (8.7)	24 (7.1)	28 (7.3)	6 (13.0)	27 (8.1)	33 (8.7)
	**Whorl**	10 (21.7)	91 (27.0)	101 (26.4)	11 (23.9)	95 (28.4)	106 (27.9)
	***P*** **. value**	0.7			0.5		
**IV**	**Loop**	22 (47.8)	139 (41.6)	161 (42.4)	20 (43.5)	149 (45.2)	169 (44.9)
	**Arch**	1 (2.2)	9 (2.7)	10 (2.6)	3 (6.5)	15 (4.5)	18 (4.8)
	**Whorl**	23 (50.0)	186 (55.7)	209 (55.0)	23 (50.0)	166 (50.3)	189 (50.3)
	***P*** **. value**	0.8			0.9		
**V**	**Loop**	30 (65.2)	246 (74.3)	276 (73.2)	29 (64.4)	251 (77.2)	280 (75.7)
	**Arch**	0 (0)	5 (1.5)	5 (1.3)	1 (2.2)	6 (1.8)	7 (1.9)
	**Whorl**	16 (34.8)	80 (24.2)	96 (25.5)	15 (33.3)	68 (20.9)	83 (22.4)
	***P. *** **value**	0.1			0.2		

Table 3 shows number and percentage of fingerprint pattern types in case and control groups. There was no significant difference in fingerprint patterns of both groups.

Table 4 represents mean TRC in case and control groups. Significant differences in mean TRC between case and control groups were found in right digit IV (*P*=0.009), left digit III (*P*=0.02), left digit IV (*P*=0.03) and left digit V (*P*=0.03). 

 The TRC asymmetry for all digits is shown in Table 5. There was no significant difference

between both groups regarding TRC asymmetry. 

 According to Table 6, significant differences were found in right hand atd angle (*P*=0.001), left hand atd angle (*P*=0.002), right hand a-b ridge (*P*=0.007) and left hand a-b ridge (*P*=0.001) between case and control group. 

 No significant differences were found in atd angle asymmetry, a-b ridge count asymmetry or pattern dissimilarity score between both groups (Table 7). *P*-values less than 0.05 were considered statistically significant.

**Table 4:** Mean TRC of CF patients and control group

** Digit**		**CF patients** **Mean Rank**	**Control group** **Mean Rank**	***P*** **. value**
**I**	**R**	156.85	197.35	0.2
	**L**	181.49	191.74	0.5
**II**	**R**	156.43	196.30	0.2
	**L**	181.49	191.74	0.5
**III**	**R**	167.84	194.15	0.1
	**L**	153.87	195.54	0.02
**IV**	**R**	150.29	195.48	0.009
	**L**	155.51	192.54	0.03
**V**	**R**	168.20	191.88	0.2
	**L**	152.44	188.30	0.03

**Table 5:** Total ridge count asymmetry for five digits in CF patients and control group

**Digit**	**CF Patients** **Mean (SD)**	**Control group** **Mean (SD)**	***P. *** **value**
**I**	193.49	190.09	0.8
**II**	206.29	187.17	0.3
**III**	178.92	190.40	0.5
**IV**	175.41	188.63	0.4
**V**	174.02	183.10	0.6

CF: Cystic Fibrosis; SD: Standard deviation

## Discussion

In this study, we observed significant differences in dermatoglyphic patterns including the mean TRC of the right digit IV, left digit III, left digit IV, left digit V and atd angle and a-b ridge in right and left hands of children with CF compared to control group.

 Kobylisky et al reported significant differences in fingerprint pattern types ^[^^17^^]^. They showed that arches fingerprint patterns were higher in CF females in contrast to higher loop patterns in CF males. Our results did not establish significant differences considering these patterns in both groups. Based on Weizman et al, whorl patterns were more frequent than loop patterns as opposed to control group in celiac patients^[^^18^^,^^19^^]^. In another study, whorl pattern values were also higher in celiac children and there was a correlation between dermatoglyphic patterns of celiac patients and their parents^[^^21^^]^.

**Table 6:** Mean (SD) for atd angles, a-b ridge counts in CF cases and control group

**Group**	**CF cases**	**Control group**	***P*** **-value**
**atd – R**	234.19	179.94	0.001^**^
**atd – L**	231.91	178.48	0.002^*^
**a-b-R**	148.75	194.59	0.007^*^
**a-b-L**	137.59	194.87	0.001^*^

SD: Standard deviation; CF: Cystic Fibrosis

In 1986, Gottlieb et al observed that the arch pattern values were significantly higher in congenital syndrome of early onset constipation and abdominal pain^[^^20^^]^. Mathew et al found increased loop patterns in children with oral cleft^[^^1^^]^. In this study, we observed significant differences in the mean TRC of the right digit IV, left digit III, left digit IV, left digit V of CF cases compared to the control group. 

 Eslami et al observed significant differences in the mean TRC of the right digit IV, right digit V and left digit II in patients with cleft lip (CLP) compared to control group^[^^3^^]^.

 This finding was in contrast to Kobylisky et al study^[^^17^^]^ which reported no significant difference in the mean TRC. Kobylisky et al also showed that mean TRC values were lower in the CF group. Their results are in line with ours^[^^17^^]^. In 2002, Neiswanger et al observed no significant differences in TRC asymmetry in patients with cleft lip who had a negative family history^[^^21^^]^. 

**Table 7:** Mean atd angle asymmetry, a-b ridge count asymmetry and pattern dissimilarity score in Cystic Fibrosis cases and control group

**Group**	**CF cases**	**Control group**	***P*** **-value**
**atd asymmetry**	196.80	180.53	0.333
**a-b asymmetry**	183.54	186.34	0.8700
**Pattern dissimilarity score**	192.93	182.75	0.529

CF: Cystic Fibrosis

Their results were similar to those of our study.

 Comparing the a-b ridge count, we found that a-b ridge count values were lower in CF patients. It was similar to Kobylisky et al study^[^^17^^]^. Rezaeinezhad et al showed that mean a-b ridge count values in patients with type 1 diabetes mellitus were higher in control group^[^^10^^]^.

 In 1973, Taussing et al observed increased atd angle in CF children, which was in agreement with our results^[^^22^^]^. On the contrary, Kobylisky et al reported that the value of atd angles were significantly lower in CF females and significantly higher in CF males^[^^17^^]^. According to the study of Mathew et al, atd angles showed an increase in children with oral cleft^[^^1^^]^.

 Esalmi et al observed that atd angles were not significantly different in CLP patients and control group. In 2013, Eslami et al observed no significant differences in a-b ridge asymmetry and pattern dissimilarity score in CLP patients, which was in agreement with our results ^[^^3^^]^.

 This study had its limitations. The limitations were confined to the small number of patients that may impose a negative effect on the final results. The authors recommend that further researches should be done in parents of CF children to assess the child and parent dermatoglyphic traits relation. Moreover, evaluating the distribution of fingerprint minutiae and palmar sweat glands in CF children would provide more comprehensive information of dermatoglyphic patterns in CF children. 

## Conclusion

Dermatoglyphic characteristics were significantly different in CF children and control group. These traits could be used as a supplementary diagnostic method in CF children.
